# Social Cooperativity of Bacteria during Reversible Surface Attachment in Young Biofilms: a Quantitative Comparison of Pseudomonas aeruginosa PA14 and PAO1

**DOI:** 10.1128/mBio.02644-19

**Published:** 2020-02-25

**Authors:** Calvin K. Lee, Jérémy Vachier, Jaime de Anda, Kun Zhao, Amy E. Baker, Rachel R. Bennett, Catherine R. Armbruster, Kimberley A. Lewis, Rebecca L. Tarnopol, Charles J. Lomba, Deborah A. Hogan, Matthew R. Parsek, George A. O’Toole, Ramin Golestanian, Gerard C. L. Wong

**Affiliations:** a Department of Bioengineering, University of California, Los Angeles, Los Angeles, California, USA; b Department of Chemistry and Biochemistry, University of California, Los Angeles, Los Angeles, California, USA; c California NanoSystems Institute, University of California, Los Angeles, Los Angeles, California, USA; d Max Planck Institute for Dynamics and Self-Organization (MPIDS), Göttingen, Germany; e Key Laboratory of Systems Bioengineering (Ministry of Education), Tianjin University, Tianjin, People’s Republic of China; f School of Chemical Engineering and Technology, Tianjin University, Tianjin, People’s Republic of China; g Collaborative Innovation Center of Chemical Science and Engineering, Tianjin University, Tianjin, People’s Republic of China; h Department of Microbiology and Immunology, Geisel School of Medicine at Dartmouth, Hanover, New Hampshire, USA; i School of Mathematics, University of Bristol, Bristol, United Kingdom; j Department of Microbiology, University of Washington, Seattle, Washington, USA; k Department of Plant and Microbial Biology, University of California, Berkeley, Berkeley, California, USA; l Rudolf Peierls Centre for Theoretical Physics, University of Oxford, Oxford, United Kingdom; m Department of Microbiology and Molecular Genetics, University of Pittsburgh School of Medicine, Pittsburgh, Pennsylvania, USA; Mass General Hospital

**Keywords:** *Pseudomonas aeruginosa*, bacterial biofilms, reversible attachment, stochastic model, surface sensing

## Abstract

What are bacteria doing during “reversible attachment,” the period of transient surface attachment when they initially engage a surface, besides attaching themselves to the surface? Can an attaching cell help any other cell attach? If so, does it help all cells or employ a more selective strategy to help either nearby cells (spatial neighbors) or its progeny (temporal neighbors)? Using community tracking methods at the single-cell resolution, we suggest answers to these questions based on how reversible attachment progresses during surface sensing for Pseudomonas aeruginosa strains PAO1 and PA14. Although PAO1 and PA14 exhibit similar trends of surface cell population increase, they show unanticipated differences when cells are considered at the lineage level and interpreted using the quantitative framework of an exactly solvable stochastic model. Reversible attachment comprises two regimes of behavior, processive and nonprocessive, corresponding to whether cells of the lineage stay on the surface long enough to divide, or not, before detaching. Stark differences between PAO1 and PA14 in the processive regime of reversible attachment suggest the existence of two surface colonization strategies. PAO1 lineages commit quickly to a surface compared to PA14 lineages, with early c-di-GMP-mediated exopolysaccharide (EPS) production that can facilitate the attachment of neighbors. PA14 lineages modulate their motility via cyclic AMP (cAMP) and retain memory of the surface so that their progeny are primed for improved subsequent surface attachment. Based on the findings of previous studies, we propose that the differences between PAO1 and PA14 are potentially rooted in downstream differences between Wsp-based and Pil-Chp-based surface-sensing systems, respectively.

## Abstract (precis)

The initial pivotal phase of bacterial biofilm formation known as reversible attachment, where cells undergo a period of transient surface attachment, is at once universal and poorly understood. What is more, although we know that reversible attachment culminates ultimately in irreversible attachment, it is not clear how reversible attachment progresses phenotypically, as bacterial surface-sensing circuits fundamentally alter cellular behavior. We analyze diverse observed bacterial behavior one family at a time (defined as a full lineage of cells related to one another by division) using a unifying stochastic model and show that our findings lead to insights on the time evolution of reversible attachment and the social cooperative dimension of surface attachment in PAO1 and PA14 strains.

## INTRODUCTION

Biofilms are surface-adhered communities or suspended aggregates of bacteria that have increased tolerance to environmental stresses and antibiotics and that impact human health and the environment in complex ways. These biofilms can be harmful by causing diseases (1, 2) and can be beneficial by serving as commensals in various hosts; they also have applications in bioremediation and energy production (3). A critical step in forming a bacterial biofilm is surface sensing (4), where free-swimming planktonic cells detect, attach to, and physiologically respond to a surface. Recent work has shown that different appendages or extracellular structures, such as flagella (5, 6) or type IV pili (TFP) (7, 8), are involved in activating cellular responses (e.g., protein production, motility, and biofilm formation) during surface sensing. In many bacterial species, these responses are controlled primarily by intracellular secondary messenger molecules, such as cyclic diguanylate (c-di-GMP) (9–16) and cyclic AMP (cAMP) (8, 17, 18). For Pseudomonas aeruginosa, a clinically relevant model system (19), there are at least two well-studied but distinct surface-sensing circuits, the Wsp and the Pil-Chp systems, which can contribute to initiating biofilm formation. In our current understanding, the Wsp system senses through the membrane-bound, chemosensory-like Wsp protein complex, which localizes laterally along the cell body (10), activating the diguanylate cyclase WspR and c-di-GMP synthesis via a mechanism that requires WspR clustering (20). On the other hand, the Pil-Chp system senses a surface through polarly localized TFP, which activate the adenylate cyclase CyaB and result in cAMP synthesis. Increased cAMP levels then induce the production and secretion of PilY1, which in turn activates the diguanylate cyclase SadC and results in c-di-GMP synthesis (17). Downstream consequences of c-di-GMP synthesis include exopolysaccharide (EPS) production and motility suppression. Different strains of P. aeruginosa, such as PAO1 and PA14, utilize these surface-sensing mechanisms to various extents. The PAO1 strain uses predominantly the Wsp system (21), leading to the surface deposition of the EPS Psl (22, 23), while PA14 uses predominantly the Pil-Chp system, leading to the suppression of surface motility (17) and production of a Pel-dominant biofilm matrix (24).

Despite the existence of diverse machineries to sense, adhere to, and proliferate on surfaces, it is commonly observed that bacteria initially seem to have a difficult time attaching to a surface, as indicated by typical flow cell studies where P. aeruginosa often takes >20 h before attaching to the surface in large numbers (25, 26). This phenomenon was first reported in the 1930s (27, 28). Using high-speed microscopy to measure the distribution of surface residence times, it was previously observed that the overwhelming majority of cells that land on the surface eventually detach, and it is only after a prolonged and variable time lag that cells begin to rapidly cover the surface (8). We stress that the low apparent probability of successful attachment is not simply a matter of cells “bouncing” off the surface. For example, during reversible attachment, it is not uncommon for cells to attach and stay long enough to divide but then subsequently detach. Moreover, the unpredictability of reversible attachment cannot be circumvented with better measurement statistics; the duration of reversible attachment of individual cells and populations always appears random and does not converge to a specific duration for the same initial conditions. The foundational question that we address here is what bacteria are doing during this period of “reversible attachment” besides attaching themselves to the surface. For example, can an attaching cell help any other cell attach to the surface? If so, does it help all cells or employ a more selective strategy to help either nearby cells (spatial neighbors) or their progeny (temporal neighbors) to attach?

The combination of defining characteristics in reversible attachment, a low probability of success, intrinsic time dependence, and structurally random outcomes suggests that the use of a stochastic model may lead to interesting answers. Here, we show that the use of an exactly solvable “divide-detach” stochastic model, designed to examine the reversible attachment behaviors of P. aeruginosa PAO1 and PA14 lineages in the form of family trees, reveals differences in their reversible attachment behaviors that suggest contrasting surface colonization strategies. Within this model, reversible attachment is described by two parameters: effective division rate and effective detachment rate. We find that reversible attachment can be understood if we analyze behavior using lineage time (the time that a lineage stays continually on the surface) rather than an experiment time, defined by the time from inoculation. Specifically, reversible attachment comprises two regimes of behavior, defined by whether cells of the lineage stay on the surface long enough to divide, or not, before detaching. For lineages that detach before dividing at all, both PAO1 and PA14 behave similarly with nearly certain lineage “extinction,” wherein the entire lineage detaches. For lineages that stay long enough to divide, PAO1 and PA14 show surprisingly different behaviors. Our model provides a framework wherein time-dependent division and detachment rates and distributions of lineages can be extracted from our experiments. For PAO1, individual lineages commit relatively quickly to a surface compared to what occurs with PA14, resulting in PAO1 displaying a steadily progressive increase of a surface cell population that is irreversibly attached (i.e., committed to forming a biofilm). For the PAO1 strain, as reported previously (21), the Wsp-based surface sensing results in early c-di-GMP-mediated EPS production that can promote attachment of a cell’s spatial neighbors. In contrast, PA14 lineages exhibit high rates of cell detachment from surfaces. However, Pil-Chp surface sensing modulates motility via cAMP and allows progeny cells to retain a memory of the surface (8), so that PA14 lineages ultimately form a planktonic population that is primed for improved surface attachment; this process thus ultimately promotes irreversible attachment and biofilm formation. Our model provides a framework for understanding the cooperative and social nature of surface attachment and for categorizing different surface colonization strategies that lead to biofilm formation, each presumably with its own advantages under different circumstances.

## RESULTS

### Two regimes of reversible attachment in PAO1 and PA14 are revealed through lineage analysis.

**FIG 1:**
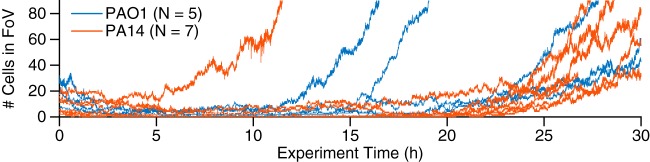
Both PAO1 and PA14 can form biofilms and have similar trends of exponential surface population increase. Each line represents one experiment for which we counted how many cells are in a single field of view (FoV) for WT PAO1 and PA14 as experiment time progresses (5 and 7 independent experiments for PAO1 and PA14, respectively). The experiment time 0 h corresponds to when imaging commenced after cells were inoculated into the flow cell chamber. Both PAO1 and PA14 have the variable lag period and the exponential increase, which is consistent with the fact that both strains initially undergo reversible attachment and then subsequently form biofilms.

When monitoring the number of cells on the surface as a function of time from the inoculation of the flow cell (denoted as the experiment time), the two strains follow similar patterns (Fig. 1). At early times, widespread detachment behavior is observed. Despite both division and additional attachment, the surface population essentially remains constant for a long and variable lag period (∼10 to 20 h), after which the surface population will then begin to rise steeply in a manner that can be fit to an exponential growth curve. However, further distinguishing their behaviors in finer detail is difficult due to the random nature of reversible attachment. When comparing the surface population increases between PAO1 and PA14, we observed nearly all possibilities: we observed either that PAO1 has a steeper and earlier rise in the surface population than PA14, that PAO1 and PA14 have similar rises, or that PA14 has an earlier and steeper rise than PAO1 (see Fig. S1 in the supplemental material). Furthermore, it is difficult to correlate these observations with macroscopic crystal violet biofilm assays, where PAO1 has statistically significantly higher optical density at 550 nm (OD_550_) values than PA14 (Fig. S2), which suggests that PAO1 is capable of forming early biofilms faster than PA14. PAO1 has a mean OD_550_ of 0.23, with a 95% confidence interval of 0.19 to 0.26, while PA14 has a mean OD_550_ of 0.14, with a 95% confidence interval of 0.099 to 0.18. Comparison of the bootstrap sampling distributions of the mean OD_550_ values (which also generate the 95% confidence intervals) shows that PAO1 has a higher mean OD_550_ value than PA14 (*P* value of 0.0002). Using the median instead of the mean gives similar results. PAO1 has a median OD_550_ of 0.22, with a 95% confidence interval of 0.17 to 0.29, while PA14 has a median OD_550_ of 0.12, with a 95% confidence interval of 0.094 to 0.21. Comparing the bootstrap sampling distributions of the median OD_550_ values (which also generate the 95% confidence intervals) show that PAO1 has a higher median OD_550_ value than PA14 (*P* value of 0.003). These apparently conflicting observations are not easily resolved with increased data collection since they arise from the intrinsic randomness of the process and not from incurring measurement errors. This intrinsic randomness, which is not uncommon in different aspects of biofilm formation, is usually neglected in analyses. In the present context, these effects complicate any analysis of the reversible attachment behaviors in PAO1 and PA14 that depend on macroscopic assays or on traditional methods to monitor the number of surface cells as a function of experiment time.

10.1128/mBio.02644-19.1 FIG S1 Exponential fits of bacteria count as a function of experiment time. (a) Each line is a fit to the function *N_e_*(*t*) = *N_e_*(0)exp[(*t* – *t*_lag_)/τ*_e_*] for the data shown in Fig. 1. *N_e_*(0) is the number of cells at time 0, *t*_lag_ characterizes the time scale of the lag period where *N_e_* is roughly constant, and τ*_e_* characterizes the time scale of exponential increase (1/τ*_e_* characterizes the rate of exponential increase). (b) Plots of τ*_e_* versus *t*_lag_ for the fits in part a. Download FIG S1, EPS file, 0.6 MB. Copyright © 2020 Lee et al. 2020 Lee et al. This content is distributed under the terms of the Creative Commons Attribution 4.0 International license.

10.1128/mBio.02644-19.2 FIG S2 Monitoring biofilm formation via crystal violet assays. The OD_550_ values are proportional to the amount of biofilm stained by crystal violet. Circles represent individual biological replicates, each of which is the mean of results from 4 technical replicates. Horizontal lines represent the mean OD_550_ values. Vertical lines indicate the 95% confidence intervals calculated from the bootstrap sampling distribution of the mean values. Download FIG S2, EPS file, 0.7 MB. Copyright © 2020 Lee et al. 2020 Lee et al. This content is distributed under the terms of the Creative Commons Attribution 4.0 International license.

**FIG 2:**
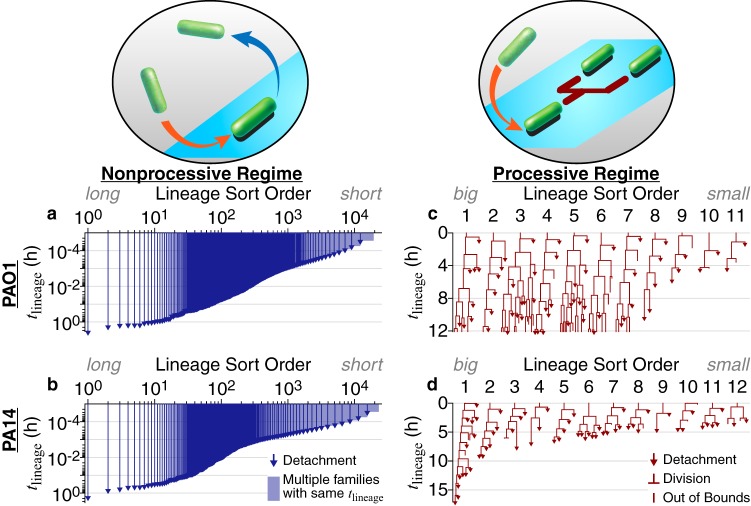
PAO1 and PA14 family trees in different regimes of reversible attachment. (a, b) Families in the nonprocessive regime of reversible attachment, which is when cells detach before dividing. Both axes are on a log scale. In this nonprocessive regime, PAO1 has *m* = 19,353 tracked families, and PA14 has *m* = 23,104 tracked families. Note the similarities between PAO1 and PA14. (c, d) Families in the processive regime of reversible attachment, which is when cells divide at least once before detaching. Both axes are on a linear scale. Each family starts at a *t*_lineage_ of 0 h, when the founder cell attaches to the surface. Tracking continues for that family until either all members detach or we lose track of the family (where we can no longer distinguish individual cells or the cells move out of the recording boundaries). We then record this time as the family’s residence time. For each regime and strain, we sort families by residence times in descending order, which sorts them by the amount of time that they have continuously contacted the surface. In this processive regime, families here are used for the model and are a subset of the fully tracked families shown in Fig. S3.

To account for the random nature of reversible attachment and the large fluctuations in the observations, we investigate the evolution of bacterial behavior as a function of surface-sensing progression using lineage analysis. We monitor the time that a given isolated family, consisting of an attached cell (founder cell) and its progeny (daughter cells) via division, stays continually on the surface, which we designate lineage time (*t*_lineage_). For each family, we begin tracking at the frame that an individual, founder bacterium attaches and assign this time as a *t*_lineage_ of 0 h. We continue tracking either until the entire family detaches or until we lose track of that family (when we can no longer distinguish individual cells or the cells move out of the recording boundaries). This final time point is recorded as the family’s residence time. During reversible attachment regimes, families are categorized by whether a division event occurs, or not, before detaching. We denote families that detach before dividing at all as the “nonprocessive” regime of reversible attachment and families that divide one or more times before detaching as the “processive” regime of reversible attachment, using language from enzyme kinetics. It is important to note that these regimes are distinct from irreversible attachment because during both regimes of reversible attachment, detachment is still prominent, while during irreversible attachment, detachment is much less common. All families analyzed here are shown in Fig. 2 and Fig. S3.

10.1128/mBio.02644-19.3 FIG S3 All families in the processive regime of reversible attachment, which is when cells divide at least once before detaching. We monitor the time that a given isolated family, consisting of an attached cell (founder cell) and its progeny (daughter cells), stays continually on the surface, which we designate lineage time (*t* = *t*_lineage_). For each family, we begin tracking at the frame individual, founder bacteria attach and assign this as a *t*_lineage_ of 0 h. We continue tracking until either the entire family detaches or we lose track of that family (moves to where we can no longer distinguish individual cells, or the cells move out of the recording boundaries). This final time point is recorded as the family’s residence time. For families of which we lose track, their true residence times can be greater than or equal to these recorded values, while for families that end in detachment, their recorded and true residence times are equal. Only families that end in detachment are used for calculating the average residence time to avoid the uncertainty in actual residence times for families of which we lose track. For each regime and strain, we sort families by residence times in descending order, which sorts them by the amount of time that they have continuously contacted the surface. Lineage indices that are boxed and bold are the families selected for use in the model. The criteria used to select families for the model are described as follows. We set the minimum number of families required for the model at ∼10 families. First, we select all families that result in lineage “extinction” events (where we observe the family ending in a detachment event), because all these families have defined outcomes (recorded and true residence times are equal). Conversely, families of which we lose track (move to where we can no longer distinguish individual cells, or the cells move out of the recording boundaries) have undefined outcomes (true residence times are greater than or equal to the recorded residence times). For PA14, this results in 12 selected families (numbers 1, 6, 9, 10, 12, 16, 19, 21, 24, 26, 28, and 31), which meets the minimum family number requirement. For PAO1, this results in 4 selected families (numbers 11, 34, 39, and 40), which is not enough. To increase the number of selected families for PAO1, we apply a residence time cutoff to the families with undefined outcomes, so that all selected families have defined outcomes at least in the lineage time window in which the analysis is performed. A residence time cutoff of *t*_lineage_ = 12 h (dashed grid line) results in an additional 7 families (numbers 1 to 7), for a total of 11 selected families for PAO1, which meets the minimum family number requirement. Download FIG S3, EPS file, 1.5 MB. Copyright © 2020 Lee et al. 2020 Lee et al. This content is distributed under the terms of the Creative Commons Attribution 4.0 International license.

Cells in both nonprocessive and processive regimes are present throughout the biofilm formation process. However, during the initial variable lag period, where the total surface population does not increase, almost all cells are in the nonprocessive regime, while very few cells are in the processive regime. As experiment time elapses, the general observed trend is that cells in the processive regime become more common, while cells in the nonprocessive regime become less common, especially during periods of surface population exponential increase. However, it is difficult to quantify such cellular behavior in this system because both regimes coexist with fluctuating proportions due to the inherent randomness in single-cell behavior, thereby complicating any analysis of biofilm behavior as a function of experiment time. Thus, we utilize an analysis of lineage time to quantify the behavior of individual families in each regime.

When comparing the two regimes for either PAO1 or PA14, we find that the residence times are drastically different. In the nonprocessive regime, ∼99% of cells stay on the surface for less than 30 s for both strains. Furthermore, of the ∼20,000 tracked families in the nonprocessive regime (both PAO1 and PA14), we observe fewer than 10 families (∼0.05%) that have residence times comparable to the average doubling time of 1 to 2 h (Fig. S4), which is the minimum residence time for families in the processive regime. Detachment dominates attachment and division in the nonprocessive regime, and essentially the surface population does not increase over the first 10 to 20 h of experiment time. In contrast, cells in the processive regime are in continuous contact with the surface for longer periods of time. Moreover, virtually all of the cells that remain surface engaged in the processive regime do so longer than cells in the nonprocessive regime. Finally, cells in the processive regime grow and divide on the surface and have clearly altered their behavior compared to that of “surface-naive” planktonic cells, presumably as a consequence of activating surface-sensing pathways.

10.1128/mBio.02644-19.4 FIG S4 Distribution of division times for PAO1 and PA14. Division time is calculated as the time between consecutively observed division events in a family. Distributions are plotted as probability density functions (pdf), calculated via kernel density estimation. PAO1 has a median division time of 1.92 h with a 95% confidence interval of 1.87 h to 1.96 h, and PA14 has a median division time of 1.42 h with a 95% confidence interval of 1.38 h to 1.45 h. PAO1 has a mean ± standard deviation division time of 1.96 ± 0.44 h, and PA14 has a mean ± standard deviation division time of 1.48 ± 0.30 h. Download FIG S4, EPS file, 0.5 MB. Copyright © 2020 Lee et al. 2020 Lee et al. This content is distributed under the terms of the Creative Commons Attribution 4.0 International license.

Interesting trends emerge when PAO1 and PA14 lineages in each regime are compared. In the nonprocessive regime, we find that PAO1 and PA14 exhibit similar behaviors, where cells experience the surface transiently. However, in the processive regime, we see stark differences between PAO1 (44 families with 622 total descendants analyzed) and PA14 (31 families with 381 total descendants analyzed) (Fig. S3). PAO1 families have more progeny stay on the surface, while PA14 families have more progeny detach, which can be seen in a broad range of metrics. For example, we can compare single-cell detachment behaviors via the proportion of detachment versus division events. PAO1 has a statistically significantly lower proportion, with 143 (33%) detachment versus 289 (67%) division events, than PA14, with 130 (43%) detachment versus 175 (57%) division events, according to the χ^2^ test (*P* value of 0.008). We can compare family-averaged detachment behaviors with the family tree asymmetry parameter (Λ) (8). Λ values close to zero indicate a more symmetric family tree in which more progeny are retained (there are more “two-legged” division nodes in the family tree, where both postdivision daughter cells stay on the surface), while Λ values close to one indicate a more asymmetric family tree where more progeny detach (there are more “one-legged” division nodes in the family tree, where one of the postdivision daughter cells detaches from the surface). PAO1 family trees have a median Λ of 0.33, with a 95% confidence interval of 0.25 to 0.39, while PA14 family trees have a median Λ of 0.42, with a 95% confidence interval of 0.37 to 0.52. Comparing the bootstrap sampling distributions of the median tree asymmetry values (which also generate the 95% confidence intervals) shows that PAO1 family trees have a lower median Λ than PA14 family trees (*P* value of 0.015). Overall, our data show that PAO1 and PA14 display similar behaviors during the nonprocessive regime, but during the processive regime, PAO1 shows a significantly higher likelihood of remaining surface associated.

### Divide-detach stochastic model highlights differences between PAO1 and PA14 in the processive regime of reversible attachment.

Our observations suggest that PAO1 is less prone to detachment than PA14. However, these metrics do not properly consider the collective time-dependent effects of division and detachment. For example, more detachment events earlier in lineage time would have a much greater effect on the resulting family architecture than the same number of detachment events occurring several generations later. Even at the single-cell level, gene expression is stochastic and can occur in a burst-like, intermittent manner (29), which contributes additional randomness to that cell’s behavior. Consequently, the behavior of an individual bacterium (in terms of whether or not in every instance it stays on the surface or detaches after a division event) may be completely random and can be described only using statistical metrics. Since biofilm formation can be seen as an evolution of a population of random individual bacteria, it can be described as a stochastic process that depends on a number of control parameters as well as random environment variables. Consistently with that contention, large fluctuations are often observed in measured parameters (e.g., family trees), and these fluctuations are not easily mitigated with increased data collection since they arise from the intrinsic randomness of the process and not from incurring measurement errors. In general, although it is acknowledged that the unpredictability of single-cell behavior can be important to surface sensing and biofilm development, this randomness is rarely accounted for in traditional microbiological studies.

**FIG 3:**
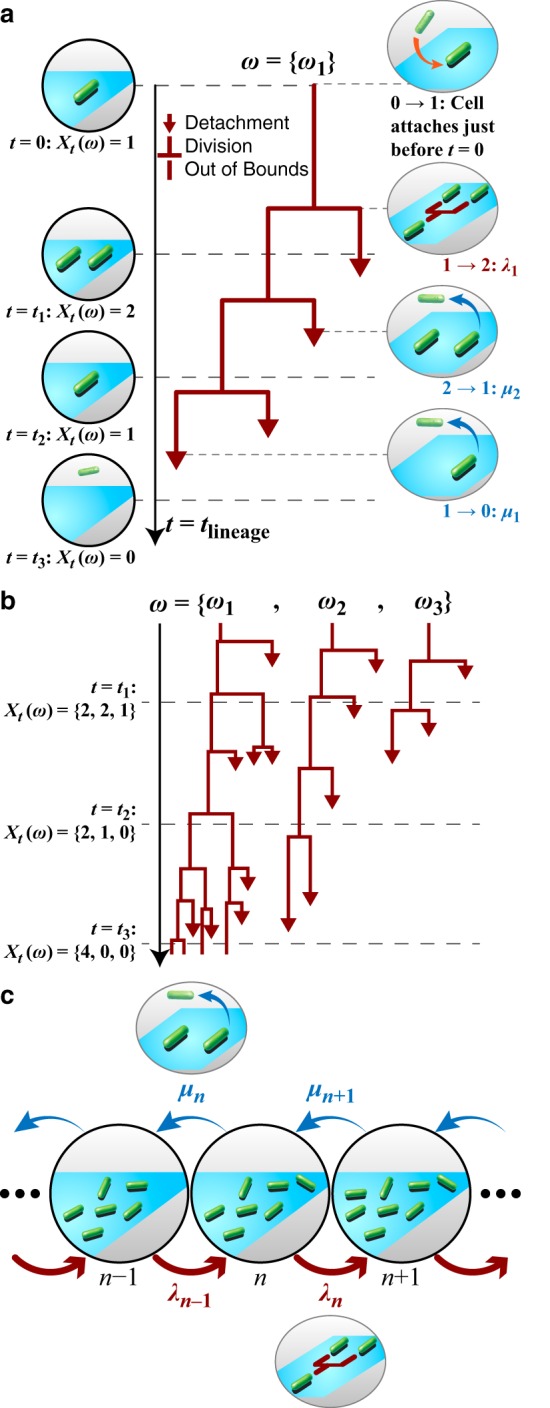
Generating family trees is a stochastic process. (a) Example illustrating the stochastic process with *m* = 1 family tree. In this case, at time *t*, *X_t_*(ω) is the number of observed cells in the family tree ω = {ω_1_}. Attachment of the founder cell happens just before *t* (*t*_lineage_) equals 0, so it is not explicitly captured by this process. When a cell divides, it undergoes a transition, *n* → *n* + 1 = λ*_n_*, and when it detaches, it undergoes a transition, *n* → *n* – 1 = μ*_n_*, where *n* is the state (i.e., the number of observed cells) before the transition. (b) Example illustrating the stochastic process with *m* = 3 family trees. At time *t*, $X_{t}\left(\omega \right)=\left\{X_{t}^{1}\left(\omega_{1}\right),X_{t}^{2}\left(\omega_{2}\right),X_{t}^{3}\left(\omega_{3}\right)\right\}$, which is the number of observed cells for each of the family trees ω = {ω_1_, ω_2_, ω_3_}. (c) Dynamics of the stochastic process for state *n*. As shown in part a, a transition (*n* → *n* + 1 = λ*_n_*) occurs when a cell divides, and a transition (*n* → *n* – 1 = μ*_n_*) occurs when a cell detaches.

To obtain more time-dependent comparisons that incorporate division and detachment effects and to help account for the inherent randomness in observed family trees, we developed a divide-detach stochastic model. We use this model to study the temporal evolution of the expected number of surface cells in a family tree, or population size (30–35). In this model, the population size can increase or decrease by one bacterium as time evolves, and the population size can be infinite or null. The corresponding sample space Ω is obtained by Ω = {0, 1, 2, …}*^m^*, where *m* is the number of independent family trees, or different populations of bacteria. As time evolves, the population size can change and result in a sequence ω ∈ Ω, where ω is the set of family trees that are in the experiment. For example, if there is *m* = 1 family tree, then ω = {ω_1_}, and if there are *m* family trees, then ω = {ω_1_, …, ω*_m_*}. However, because this is a stochastic process, we cannot predict ahead of time what ω will be. Instead, what we know for ω is the set of possible observations or states Σ and the actual observations *X_t_* from experiments. The set of states is given by Σ = {0, 1, …, *N*, …}, where *N* represents the number of surface cells in a family and is infinite. Σ tells us what observations (number of surface cells) are possible for any family tree during an experiment. The actual observations of ω are given by $X_{t}(\omega )=\left\{X_{t}^{1}(\omega_{1}),X_{t}^{2}(\omega_{2}), . . .,X_{t}^{m}(\omega_{m})\right\}$, which is how many surface cells are observed in each of the *m* family trees at time point *t* during an experiment, and *X_t_*(ω) is a random variable *X_t_*: ω → Σ that defines this stochastic process. Having a random variable means that for the family trees ω and each time point *t* we observe *X_t_*(ω) taken from the set of states Σ according to a certain (not necessarily known) probability distribution, but when we repeat the experiment, we will not necessarily observe the same *X_t_*(ω) for the same time point *t* and family trees ω. Figure 3 shows examples of this process for *m* = 1 family tree (Fig. 3a) and for *m* = 3 family trees (Fig. 3b).

The dynamics of such a stochastic process are given by the evolution of the probability distribution *P*(*j*,*s* + *t*|*i*,*s*), which gives the probability of transitions between all states, *i* and *j*, and for all times *s* and *s* + *t* that are ≥0, and the probability of transitions can be rewritten as *P_t_*(*j*|*i*). For a family tree, the only possible transitions are the neighboring transitions indicated with the equations *n* → *n* + 1 = (*n* + 1|*n*) = λ*_n_* and *n* → *n* – 1 = (*n* – 1|*n*) = μ*_n_*. As a result, the dynamics of this process can be described by looking only at the evolution of the probability distribution, *P_t_*(*n*|*n*), for state *n* ∈ Σ. The rates λ*_n_* and μ*_n_* determine the intensity of increase (i.e., division) or decrease (i.e., detachment), respectively, for state *n*. In a family tree, each cell can divide (with a division rate, λ) or detach (with a detachment rate, μ), so that the rates become λ*_n_* = λ*n* and μ*_n_* = μ*n*. Figure 3c shows a schematic of the dynamics described here (i.e., how the population size can increase or decrease).

The equation describing the evolution of this process is given by the Kolmogorov forward equation, also called the master equation, which reads

(1) $$\begin{array}{l} \frac{dP_{0}}{\mathrm{dt}}=\mu P_{1},n=0,\text{ and }\\ \frac{dP_{n}}{\mathrm{dt}}=\left(\mu \right)\left(n+1\right)P_{n+1}-\left(\lambda +\mu \right)\left(n\right)P_{n}+\left(\lambda \right)\left(n-1\right)P_{n-1},n>0, \end{array}$$

where $P_{n}=P_{n}\left(t\right)=P_{t}\left(n\text{|}n\right)$, $P_{n+1}=P_{n+1}\left(t\right)=P_{t}\left(n+1\text{|}n\right)$, and $P_{n-1}=P_{n-1}\left(t\right)=P_{t}\left(n-1\text{|}n\right)$. We refer the reader to Materials and Methods to find the details of the solution to this equation.

Experimentally, by having access to *m* independent family trees, it is possible to build the probability distribution by counting the number of families that have zero cells, one cell, two cells, and so on, at a given lineage time *t*. In other words, for each time point *t*, we plot the actual observations *X_t_*(ω) on a histogram to derive the probability of each of the states *n* occurring. For families in the processive regime of reversible attachment, we avoid potential problems arising from tracking limitations by selecting *m* = 11 families for PAO1 (out of 44 families) and *m* = 12 families for PA14 (out of 31 families), with a *t*_lineage_ range of 0 to 12 h for PAO1 and 0 to 10 h for PA14 (see Fig. S3’s caption for family selection criteria).

Comparing the experimental data with the model is not straightforward when using the probability distributions directly. In the experimental data, there are a finite number of families and a finite number of cells in a family, which means that it is difficult to generate distributions that are well populated for quantitative comparisons. To overcome this limitation, we employ the method of moments, which provides information about the distributions, to fit the model to experimental data and obtain the rates. Instead of comparing the experimental and model probability distributions, *P_n_*(*t*), we compare the experimental and model moments, 〈*n*(*t*)*^k^*〉, where *k* is the *k*-th moment. We can calculate the experimental moments directly from the experimental probability distribution, and we can obtain the model moments from the model probability distribution (see equation 2 in Materials and Methods) given by the master equation (equation 1). The equations for the model moments are shown in Materials and Methods (see equations 3 and 4). To compare experiment with model, we use the first two moments. The first moment is the mean, and the second moment is related to the variance, since the variance equals the second moment minus the first moment squared.

**FIG 4:**
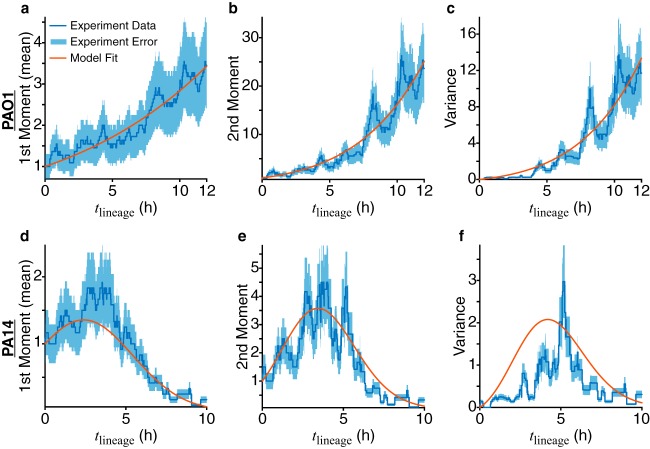
Obtaining division (λ) and detachment (μ) rates by fitting experimental and model moments of the number of cells in a family for families in the processive regime of reversible attachment. Moments and variances calculated from experimental data are plotted as blue lines, with the relative error (calculated as $1/\sqrt{m}$, where *m* is the number of families used) shown as the light-blue shaded area. Variance is defined as the second moment minus the first moment squared. Red lines show the fits to the first and second moments (equations 3 and 4) using nonlinear least-squares regression. For the model, we use the linear functional form of the rates, λ(*t*) = *L*_0_ + *L*_1_*t* and μ(*t*) = *C*_0_ + *C*_1_*t*. The resulting coefficients from the fits are as follows: for PAO1, *L*_0_ = 0.136 h^−1^, *L*_1_ = 0 h^−2^, *C*_0_ = 0.0242 h^−1^, and *C*_1_ = 0.00147 h^−2^, and for PA14, *L*_0_ = 0.256 h^−1^, *L*_1_ = 0 h^−2^, *C*_0_ = 0 h^−1^, and *C*_1_ = 0.107 h^−2^.

When we plot the moments calculated from the experimental data for families in the processive regime of reversible attachment (Fig. 4), striking differences between PAO1 and PA14 are revealed. PAO1 follows an exponential growth curve, while PA14 follows a Gaussian curve. These curves are consistent with what we see in the family trees. For PAO1, many of the families have increasing numbers of cells, while for PA14, fewer of these families are present, and most families end in detachment. However, as we have previously shown, PA14 cells that detach have already initiated the surface-sensing process, and they retain memory of the surface based on their prior surface residence, which primes them for subsequent irreversible attachment (8). Also, from our data, the variances for both PAO1 and PA14 can be as large as the mean population size, indicating that extinction in an individual lineage can happen at any time, even in a population that is exponentially growing on average. Therefore, it is important to note that individual lineage extinction events (where the entire family detaches) do not indicate a failure to form a biofilm.

With our model, the temporal evolution of a family tree can be described by the single-cell division (λ) and detachment (μ) rates. λ is likely related to cellular events that contribute to surface growth, which can be affected by complex factors, such as changes in cellular metabolism or the local availability of nutrients. Likewise, μ is likely to be related to cellular events that contribute to detachment, such as the production of EPS and the activities of motility appendages. The most general way of accounting for these complex effects is to have both rates be time dependent, so λ is equal to λ(*t*) and μ is equal to μ(*t*). For example, as bacteria continue proliferating on the surface, they can produce more EPS and start detaching less, and they can start to slow down their growth if they become nutrient limited (i.e., their nutrient consumption rate exceeds the environment’s nutrient replenishment rate). However, finding the exact functional form of time dependence to use in the model is difficult. We first start with the simplest form of time dependence (linear, or first-order polynomial), where λ(*t*) = *L*_0_ + *L*_1_*t*, μ(*t*) = *C*_0_ + *C*_1_*t*, and {*L*_0_, *L*_1_, *C*_0_, *C*_1_} are the coefficients that we obtain by fitting the experimental data to the model. λ(*t*) and μ(*t*) are rates that represent probabilities per time unit, which means that they are positive and have dimensions of inverse time ([λ] = [μ] = [time]^–1^). Therefore, the coefficients *L*_0_ and *C*_0_ also have dimensions [*L*_0_] = [*C*_0_] = [time]^–1^, and the coefficients *L*_1_ and *C*_1_ have dimensions [*L*_1_] = [*C*_1_] = [time]^–2^. By dimensional analysis, we can extract time scales for lineage-level growth (via division) and death (via detachment) behaviors from either the rates (λ^–1^ and μ^–1^) or the coefficients (*L*_0_/*L*_1_ and *C*_0_/*C*_1_). If the experimental data and model do not show good agreement, then we can reiterate this process with progressively more complicated functions. Additionally, the shape of the experimental moments can guide us in choosing the correct function for the rates.

With linear time dependence, we already obtain good agreement when fitting using nonlinear least squares, as shown by the results of the model fits to the experimental moments in Fig. 4. To ensure the fit results give meaningful coefficient values, we set the following constraints based on experimental data. The rates are positive, so λ(*t*) is greater than 0 and μ(*t*) is greater than 0. As seen in the family trees in Fig. 2 and Fig. S3, division events are roughly evenly spaced out in time, and cells are not nutrient limited inside the experimental system, so λ(*t*) should be constant. Thus, we set *L*_1_ as equal to 0 and λ(*t*) as equal to *L*_0_. We consider any coefficient that is <10^–5^ as 0 for subsequent analysis based on the precision of the experimental data. The resulting coefficients from the fits are as follows: for PAO1, *L*_0_ equals 0.136 h^−1^, *L*_1_ equals 0 h^−2^, *C*_0_ equals 0.0242 h^−1^, and *C*_1_ equals 0.00147 h^−2^, and for PA14, *L*_0_ equals 0.256 h^−1^, *L*_1_ equals 0 h^−2^, *C*_0_ equals 0 h^−1^, and *C*_1_ equals 0.107 h^−2^.

We find that μ(*t*) is time dependent for both strains (i.e., *C*_1_ is not zero). For PAO1, we find that μ slowly increases, since *C*_1_ is ∼1 order of magnitude smaller than *C*_0_, and both coefficients are positive and smaller than *L*_0_. For PA14, μ increases quite rapidly, since *C*_1_ is positive and is much greater than *C*_0_. Because μ(*t*) is time dependent, the relevant time scale (τ_μ_) to extract for time-dependent lineage-level detachment behavior for both PAO1 and PA14 is *C*_0_/*C*_1_ (≈16 h for PAO1 and 0 h for PA14). Also, because λ(*t*) is constant, the relevant time scale (τ_λ_) to extract for time-independent lineage-level division behavior for both PAO1 and PA14 is $L_{0}^{-1}$ (≈7 h for PAO1 and ≈4 h for PA14). These values are consistent with the experimental data. Interestingly, for both strains, we find that τ_λ_ is bigger than the average division time by a factor of ∼3, which means that τ_λ_ corresponds to ∼3 generations of division. The value of τ_λ_ corresponds closely to the time that a given lineage persists on the surface before ultimately going extinct and detaching (i.e., residence time). To calculate the mean residence times of the experimental lineages in Fig. 2, we include only lineages that ultimately detach before the cutoff time (12 h for PAO1 and 10 h for PA14). This results in ≈6 h for PAO1 and ≈5 h for PA14, which are very close to the τ_λ_ values obtained from the coefficients (≈7 h for PAO1 and ≈4 h for PA14 [see above]). For PAO1, having a larger τ_λ_ and a slowly increasing and relatively small μ means that lineages are division dominant (λ > μ) as they spend more time on the surface. Rather than ultimately detaching, we see many lineages persist on the surface and increase their numbers of cells despite having detachment events. At a *t*_lineage_ of 12 h, 7 of the 11 families still exist on the surface (Fig. 2c, numbers 1 to 7). For PA14, having a smaller τ_λ_ and a rapidly increasing μ means that lineages are initially division dominant (λ > μ) but then become detachment dominant (μ > λ) after a certain amount of time on the surface, which is also the time scale described by τ_λ_. We see that many lineages grow to at least 2 to 3 generations, which corresponds to the lineage time where λ is greater than μ. Once μ is greater than λ, then many families begin to detach until, at a *t*_lineage_ of 10 h, only 1 of the 12 families still exists on the surface (Fig. 2d, number 1). Clearly, unlike PAO1, PA14 cells that have started the surface-sensing process do not necessarily stay on the surface. Rather, as we have shown previously (8), they rejoin the planktonic population as “surface-sentient” cells that are primed for longer surface residence times during subsequent attachment. Similarly, the value of τ_μ_ corresponds closely to cellular activities that affect detachment, such as, for example, the competition between EPS production and motility appendage activity. EPS is likely to affect detachment more for PAO1 than for PA14, since PAO1 is known to produce the Psl EPS, while PA14 cannot. On the other hand, presumably because of the Pil-Chp system, motility appendage activity is likely to affect detachment more for PA14 than for PAO1. Consistently with this hypothesis, PAO1 has a much larger τ_μ_ than PA14 (which is zero).

**FIG 5:**
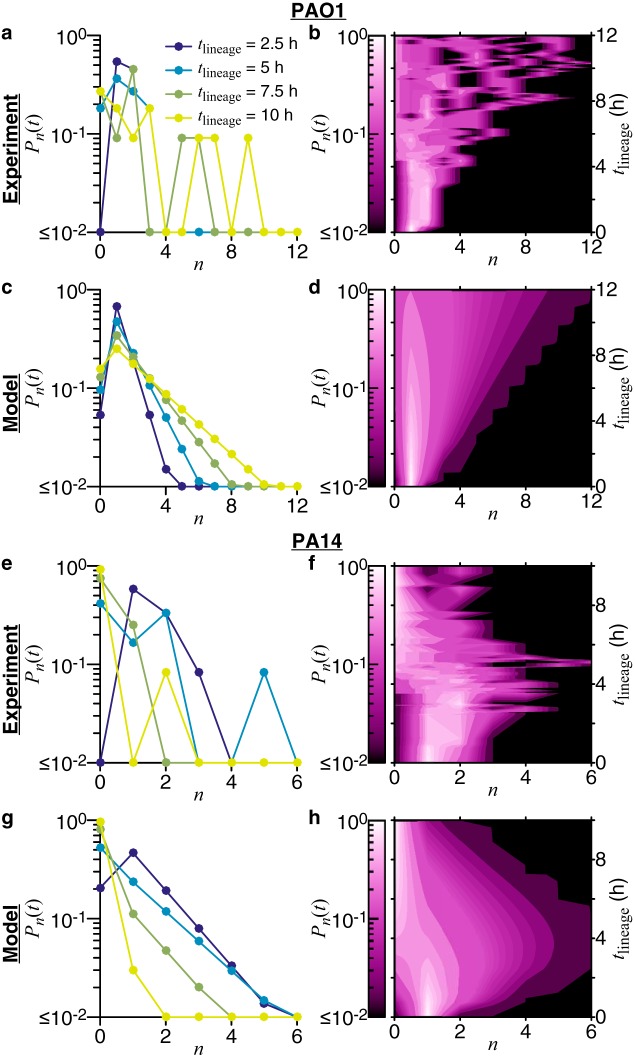
Comparing experimental and model probability distributions for the number of cells in a family for families in the processive regime of reversible attachment. Experimental probability distributions are built directly from the data, as described in Materials and Methods (in the section “Multigenerational-family tracking analysis”). For the model probability distribution *P_n_*(*t*), we use equation 2 and the linear functional form of the rates, λ(*t*) = *L*_0_ + *L*_1_*t* and μ(*t*) = *C*_0_ + *C*_1_*t*. The model rate coefficients used are shown in Fig. 4. Probability distributions are compared in two different ways. The left column shows plots of *P_n_*(*t*) versus *n* for specific lineage times of *t* = {2.5, 5, 7.5, 10} h, and the right column shows plots of the entire *P_n_*(*t*) versus *n* and *t*, where the probability is represented by the shades of color in the contour plots. Probabilities are shown on a log scale.

With these model parameters, we can evaluate the model probability distribution, *P_n_*(*t*), to then compare with the experimental probability distribution, *P_n_*(*t*). We show the comparisons of the probability distributions with two different visual representations in Fig. 5. The first plots *P_n_*(*t*) versus *n* for specific lineage times (*t* = {2.5, 5, 7.5, 10} h), and the second plots the entire *P_n_*(*t*) versus *n* and *t* as a contour plot. The plots of the probability distributions also show good agreement. For the probability contour plots, agreement between experiment and model are assessed as follows. The regions of high and low probability contours [*P_n_*(*t*) is ∼1 and *P_n_*(*t*) is ≤10^–2^, respectively] are similar in shape and location (in the *n*, *t* plot space) between experiment and model. The shapes of the probability contours are consistent with the plots of the moments in Fig. 4. For PAO1, as *t* progresses, the probability of having more cells per family (higher *n*) increases. For PA14, the probability of having higher *n* increases and then decreases as *t* progresses. However, further direct comparisons of the probability distributions are difficult. As previously mentioned, the experimental probability distributions will invariably be sparser than the model probability distributions, which can be seen in the plots as either jagged lines or holes in the contours. This sparseness comes from having finite experimental data and is not straightforward to remove (e.g., via interpolation). Nevertheless, the model probability distribution can be used to describe what family tree architectures we expect to observe during similar experiments.

**FIG 6:**
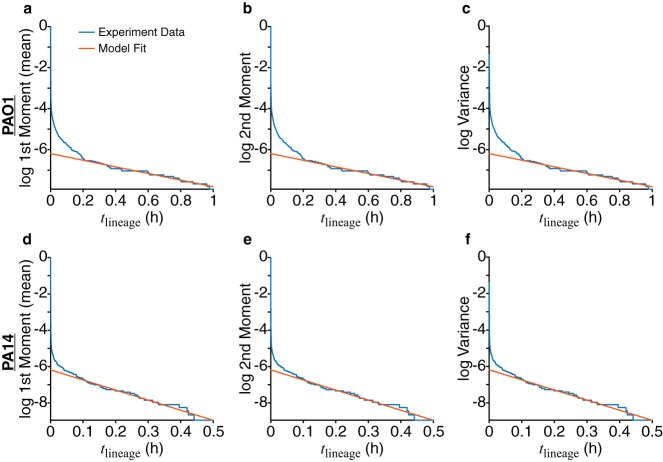
Fitting experimental moments show that families in the nonprocessive regime of reversible attachment have only detachment events. The natural logarithm (log) of the moments and variances calculated from experimental data are plotted as blue lines. Relative errors (calculated as $1/\sqrt{m}$, where *m* is the number of families used) are not plotted here, as *m* is approximately 20,000 for both PAO1 and PA14, and the values are very small. Red lines show the fits of the experimental data to the function {log[*y*(*t*)] = *a*_1_*t* + *a*_0_}. For PAO1, fits were performed for 0.2 h ≤ *t* ≤ 1 h, with the resulting fit coefficients as *a*_1_ = –1.61 h^−1^ and *a*_0_ = –6.19. For PA14, fits were performed for 0.1 h ≤ *t* ≤ 0.5 h, with the resulting fit coefficients as *a*_1_ = –5.55 h^−1^ and *a*_0_ = –6.19.

The model can also be applied for cells in the nonprocessive regime of reversible attachment. We find that for both PAO1 and PA14, the moments fit an exponential decay function (Fig. 6). This is what the model predicts if there is only detachment and no division, and it correctly describes the data, because cells in the nonprocessive regime detach before dividing on the surface. Furthermore, the variances are of the same order of magnitude as the mean population, which means that lineages can become extinct at any time. Thus, for both processive and nonprocessive regimes of reversible attachment, the stochastic model described here accurately describes the behaviors of PAO1 and PA14, including their differential paths to irreversible attachment.

### PAO1 and PA14 have distinct progressions of surface attachment, which suggest contrasting surface colonization strategies.

Our observations and results imply that both PAO1 and PA14 start their initial surface engagement with similar behaviors (in terms of surface residence times) in the nonprocessive regime of reversible attachment, but then they diverge strongly in the processive regime of reversible attachment. PAO1 shows an increase in the number of families that commit relatively quickly to surface growth, and this is likely a factor that contributes to the trend of PAO1 forming faster biofilms than PA14, as seen in the crystal violet assays (Fig. S2). For PAO1, this early attachment behavior can be more intuitive when correlating with the general progression of biofilm formation. PA14, in contrast, shows a larger number of detachment-dominated families even though the entire population eventually forms a biofilm, which can be a counterintuitive result. Compared to PAO1, where production of sticky EPS appears to be the dominant mechanism driving irreversible attachment (21–23), PA14 appears to utilize a different surface colonization strategy dependent on progressive suppression of surface motility appendage activity (8). While the production of EPS facilitating biofilm formation for PAO1 is in line with current models for biofilm formation (21–23), it is not obvious how this motility suppression strategy in PA14 can lead to rapid changes in bacterial detachment rates from surfaces, as shown by the model.

**FIG 7:**
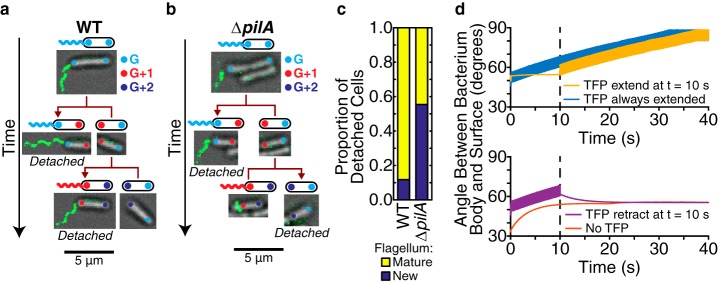
Both TFP and flagella are important for the detachment process in PA14. (a, b) The location of the flagellum and the age of poles (measured in generations) can be tracked across multiple generations. The fluorescence image of the labeled flagellum is laid on top of the bright-field image, and the poles are overlaid as colored circles (color representing the pole generation *G*). Scale bars for the pictures are 5 μm. (a) Example tracking for the WT, where cells with mature flagella detach. (b) Example tracking for the Δ*pilA* mutant (with deletion of the major subunit for the TFP filament), where one cell with a mature flagellum and one cell without a flagellum detach. (c) Proportion of detached cells with a mature flagellum versus a new flagellum (which includes no flagellum), calculated from 154 events for the WT and 74 events for the Δ*pilA* mutant. The proportions are statistically significantly different between the strains according to the χ^2^ test (*P* value is much less than 10^–4^). (d) Angles that the bacterium's body makes with the surface under different TFP conditions in the hydrodynamic model: with TFP extension at a *t* of 10 s (top plot, yellowish orange), with TFP retraction at a *t* of 10 s (bottom plot, purple), with TFP extended throughout (top plot, blue), and with no TFP (bottom plot, red). If the bacterium does not spin, then the angle between the body and surface will stay under the (arbitrary) initial condition that we have chosen in the model. We show time using units of seconds and a torque value of 2 pN μm (37).

We focused on detachment events for PA14 to gain insight into this alternate surface colonization strategy and why appendages and their activities can give rise to a time-dependent detachment rate, μ. Consistently with previous results where flagellum-mediated surface spinning generally results in a detachment event (36), we find that ∼90% of detachment events occur when a cell has the mature flagellum inherited from its ancestor, as opposed to that cell having to form a new flagellum postdivision (Fig. 7a and c). Interestingly, deleting the *pilA* gene (Δ*pilA*, causing the lack of the major subunit of the TFP filament) results in significantly fewer detachment events (χ^2^ test *P* value is much less than 10^–4^) for cells that have a mature flagellum. Compared to the wild type (WT), only roughly half of detachment events occur when the cell has a mature flagellum in the Δ*pilA* mutant (Fig. 7b and c), an observation that suggests that TFP are important to the detachment process. For the Δ*pilA* mutant (and to a much lesser extent in the WT), we also observed detachment events with cells that did not have a labeled flagellum, which suggests that non-flagellum-mediated detachment events can also occur.

To study how TFP can influence flagellum-mediated spinning and detachment, we adapt a previously developed hydrodynamic model (37). Simulations show that TFP activity (i.e., extension or retraction) can lead to changes in the cell body tilt angle relative to the surface. In the case where the nonflagellated pole is attached to the surface, TFP extension during flagellum-mediated spinning results in the cell tilting to nearly vertical orientations, while retraction results in a smaller tilt angle (Fig. 7d). During flagellum-mediated spinning, nearly vertical orientations correlate with higher rates of detachment, while orientations closer to the surface correlate with a decreased likelihood of detachment (36, 38). Consistently with previous results, the cell without TFP is more likely to assume an orientation closer to the surface (i.e., horizontal), while the cell with TFP extended the entire time is more likely to assume a nearly vertical orientation (8).

**FIG 8:**
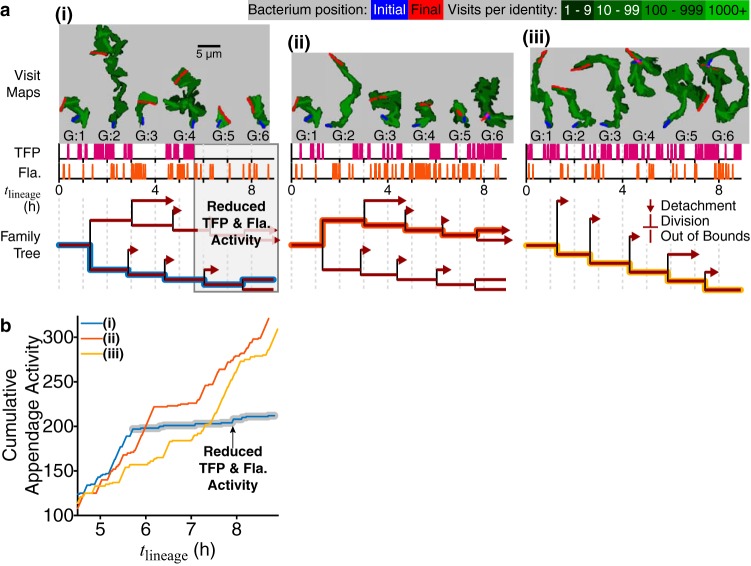
Family tree architecture controlled by generation-dependent motility activity. (a) TFP and flagellum activities are plotted for single branches of a family as magenta and red spike plots, respectively, with each spike representing one instance of activity. The colored line on the family tree plot traces the analyzed branch. We plot the corresponding visit map of each single cell (top, gray boxes), shown as shades of green in a logarithmic color scale with initial (final) positions shown in blue (red), to show the trajectory history and visual representation of TFP and flagellum (Fla.) activity. The size of each visit map is roughly proportional to the level of activity: cells with high TFP activity have elongated visit maps, while cells with high flagellum activity have circular visit maps. (i) For the WT, we see one example where TFP and flagellum activity are reduced and do not coincide, resulting in a division event where no daughter cells detach. (ii, iii) The other examples show either higher activities or activities that coincide, resulting in a continuation of division events with detachment occurring. (b) Plot of the cumulative appendage activity (i.e., the cumulative number of instances of either TFP or flagellum activity) for the 3 examples in panel a. For example i, the curve plateaus out at a *t*_lineage_ of ∼6 h, which is when the reduction in appendage activity occurs. For examples ii and iii, the curves continue to rise, which shows that the appendages continue to be active.

These results suggest that detachment rates are higher when TFP activity and flagellum activity are high and/or coincide and that detachment rates are lower when the activities are reduced and/or do not coincide. Given that PA14 has a small average family size, small surface residence times, and large surface detachment rates, observations of suppression of both appendage activity and detachment are expected to be extremely rare during reversible attachment (i.e., while cells are transiently on the surface). Nevertheless, in our family tree data, we can find examples where we can compare cells from the same generation but on different branches of the family tree. In these cases, we observe detachment in branches where appendage activities are high (and/or coincide) and no detachment in branches where appendage activities are reduced and/or do not coincide (Fig. 8). In Fig. 8, example i, we see that appendage activity is reduced around a *t*_lineage_ of ∼6 h, which coincides with the presence of a division event where no daughter cells detach. In Fig. 8a, examples ii and iii, appendage activity does not become quiescent and detachment continues to occur for subsequent division events. This appendage activity analysis was repeated with the Δ*pilA* mutant for validation, and results were consistent with previous results (Fig. S5).

10.1128/mBio.02644-19.5 FIG S5 Appendage activity tracking for the Δ*pilA* mutant. We repeat the analysis whose results are shown in Fig. 8 for the Δ*pilA* mutant and find one predominant behavior for the 9 generations shown here, consistent with this strain having predominantly one-legged division branching (8). We observe no TFP activity, which is consistent with this strain having no TFP. We observe sporadic, but prolonged, flagellum activity, which is consistent with observations of this strain spinning on the surface for prolonged periods of time, and we observe detachment events without flagellum activity, which is consistent with observations of detachment events where cells did not have a labeled flagellum. Beyond generation 9, the Δ*pilA* mutant continues to have mainly one-legged division branching for multiple subsequent generations. Download FIG S5, EPS file, 1.5 MB. Copyright © 2020 Lee et al. 2020 Lee et al. This content is distributed under the terms of the Creative Commons Attribution 4.0 International license.

## DISCUSSION

Clearly, the application of stochastic models can be quite powerful in understanding microbiological systems that involve strong fluctuations. The behavior of each lineage is a record of how a specific cell and its progeny managed to stay and proliferate on the surface during cellular changes induced by surface sensing, which has multigenerational consequences. Even though the probability of a specific cell attaching to a surface and proliferating successfully is initially vanishingly small, surface sensing can modify outcomes by changing the structure of family trees, as we can see from the evolution of reversible attachment from the nonprocessive to processive regimes, for example. Interestingly, that the process of reversible attachment can be described by a stochastic model is telling: whether a bacterium encountering a surface makes it to irreversible attachment and eventually participates in biofilm formation may be quantitatively cognate to the description of whether patient zero’s disease will die out after a few infections or take hold and become an epidemic. The fact that biofilm formation seems to inevitably happen is due to factors such as the large number of lineages that encounter the surface and the existence of multigenerational memory, which can mitigate against initial failure to attach by conditioning a planktonic population primed for improved subsequent attachment.

Indeed, a recent study applied a variation of our approach to antibiotic treatment of bacteria (39). In fact, the quantitative evolution of bacterial populations in early biofilm formation is analogous to a time-reversed version of antibiotic treatment: the nonprocessive regime of reversible attachment behaves like bacterial population dynamics for antibiotic treatment well above the MIC. In the present study, however, we are able to perform an unprecedented level of longitudinal comparison between theory and experiment. Because we have information on the fates for every cell in a large number of bacterial lineages that occur during early biofilm formation, we can directly measure and analyze the time evolution of the system. This analysis provides a conceptual framework for understanding the taxonomy of surface colonization strategies and reveals an unanticipated difference between PAO1 and PA14 behavior.

One of the old questions about biofilm formation is whether it is the newly landed cells or the dividing cells on the surface that contribute more to the biomass increase in the biofilm. Our results suggest that not only is the answer species and strain dependent, the question itself is misleading because of the assumed either/or format of the answer. Surface sensing can evolve progenitor cells which land on a surface and commit almost their entire division lineage to the surface, thereby drastically increasing biomass. Furthermore, our results suggest that surface attachment and reversible attachment can have a social dimension; when bacteria attach to the surface, they can help other cells in the population to attach and remain adhered, as described below.

### Attachment of PAO1 promotes attachment of neighboring cells; attachment of PA14 promotes attachment of progeny cells.

The divide-detach stochastic model highlights two distinct but complementary strategies for surface colonization that are illustrated by PAO1 and PA14. For PAO1, surface population increase takes the form of the few families that are more successful in retaining surface progeny. PAO1 families generally stay on the surface during biofilm formation, likely due to the Wsp surface-sensing system and early Psl EPS secretion, which facilitates surface attachment of a cell’s spatial neighbors. Previous work has shown that early surface attachment behavior depends on EPS production via the Wsp system (9, 23). In contrast, for PA14, surface population increase takes the form of many families that are less successful in retaining surface progeny due to surface detachment. However, PA14 cells and their progeny can “remember” the surface due to the Pil-Chp system and multigenerational cAMP-TFP memory (8), which primes them for biofilm formation whether they are currently on the surface or not and eventually leads to progressive suppression of motility appendage activity. Both strategies are viable for surface colonization. PAO1 cells tend to attach, increase their surface population more quickly, and persist longer on a surface than PA14 cells, which suggests that PAO1 can potentially attach to surfaces even in ecologically crowded environments or successfully form biofilms by outgrowing competing species. Indeed, this has been experimentally observed: EPS-producing P. aeruginosa strains tend to persist on surfaces better than non-EPS-producers, despite possible exploitation by “cheaters” that can potentially use the communal good of EPS (40). In contrast, PA14 cells exposed to a surface do not initially stay on the surface and slowly increase surface coverage. Rather, they and their progeny form a surface-sensing-activated planktonic population that can quickly attach and colonize the surface later in time, which may be better adapted for overwhelming host defense (i.e., a naive surface) than for microbial competition. Moreover, it is interesting to note that EPS secretion is extracellular and can be shared both with neighbors from different lineages and with descendants in close proximity to help them attach and remain attached (41). On the other hand, memory is intracellular and can be passed down only temporally through division, thus allowing cells to help only their progeny to remain attached.

It is possible that our observations and results with PAO1 and PA14 may be generalizable to other P. aeruginosa strains. The majority of strains in the International *Pseudomonas* Consortium Database (IPCD) can be identified as either PAO1-like or PA14-like based on their phylogeny (i.e., have the same phylogenetic subgroup as either PAO1 or PA14) (42–45). Consistently with our results, crystal violet biofilm assays show that the PAO1-like strains seem to produce early biofilms faster than the PA14-like strains (Fig. S6). Although it is clear from the data spread that there is more to explaining differences in biofilm behavior than pseudomonad phylogenetic diversity, this observation suggests that the phylogenetic distance from either PAO1 or PA14 may be incorporated into a metric for categorizing a P. aeruginosa strain’s biofilm formation behavior as either PAO1-like or PA14-like. It is likely that these bacterial strategies have their own advantages under different circumstances. Furthermore, our model can be applied to other bacterial systems to understand how they utilize their cellular machinery for orchestrating different types of social cooperativity during surface attachment and for their implicit surface colonization strategies.

10.1128/mBio.02644-19.6 FIG S6 Crystal violet biofilm assay results for 35 P. aeruginosa strains (25 PAO1-like and 10 PA14-like strains, including PAO1 and PA14 strains) in the International *Pseudomonas* Consortium Database (IPCD). These strains are identified as either PAO1-like or PA14-like based on their phylogeny (i.e., in the same phylogenetic subgroup as either PAO1 or PA14) (42–45). The OD_550_ values are proportional to the amount of biofilm stained by crystal violet. Circles represent individual biological replicates, each of which is the mean of results from 4 technical replicates. Longer horizontal lines represent the mean OD_550_ values. Vertical lines and error bars indicate the 95% confidence interval calculated from the bootstrap sampling distribution of the mean OD_550_ values. A comparison of these distributions shows that the mean OD_550_ values for the PAO1-like strains are higher than the mean OD_550_ values for PA14-like strains (*P* value of 0.02). Download FIG S6, EPS file, 0.6 MB. Copyright © 2020 Lee et al. 2020 Lee et al. This content is distributed under the terms of the Creative Commons Attribution 4.0 International license.

## MATERIALS AND METHODS

### Strains and growth conditions.

Pseudomonas aeruginosa PAO1 and PA14 wild-type (WT) strains were used in this study. For the flagellum localization data, WT PA14 and a Δ*pilA* mutant (with deletion of the major subunit of the TFP filament) (46) with FliC (the major subunit of the flagellum filament) modified to FliC(T394C) (47) were used. PAO1 was cultured as previously described (21, 23), and PA14 was cultured as previously described (8). Culturing protocols are summarized as follows. Bacteria were plated on LB agar plates and incubated at 37°C overnight. Individual colonies were swabbed from the plate and grown overnight for ∼18 h in an incubator at 37°C with shaking at 220 rpm. Overnight cultures were regrown under the same overnight growth conditions to an OD_600_ of ∼0.4 to 0.6. Regrowth cultures were then diluted under flow cell conditions to an OD_600_ of ∼0.01 to 0.03. These final diluted cultures were used for injection into the flow chamber.

Different medium conditions were chosen for PAO1 and PA14 based on the medium optimized for flow cell early biofilm formation experiments for each individual strain in prior work. For PAO1, overnight and regrowth medium consisted of fastidious anaerobe broth (FAB) medium with 30 mM glutamate, while flow cell medium consisted of FAB medium with 0.6 mM glutamate (21, 23). For PA14, overnight and regrowth medium consisted of M63 medium with 1 mM magnesium sulfate, 0.2% glucose, and 0.5% Casamino Acids (CAA), while flow cell medium consisted of M63 medium with 1 mM magnesium sulfate, 0.05% glucose, and 0.125% CAA (8, 46). For flagellum staining experiments, the flow cell medium also contained 0.375 μg/ml Alexa Fluor 488 C5 maleimide dye (Molecular Probes). For more details on the culturing procedures, please refer to the corresponding references. PAO1 experiments were repeated with the PA14 medium conditions, and the same basic trends discussed in this paper still held.

### Crystal violet biofilm assays.

Biofilm assays were performed as previously described, with minor modifications (48, 49). Briefly, culture inocula were grown in 100 μl of LB medium at 37°C in a 96-well microtiter plate for ∼16 h. Cultures were normalized and diluted ∼1:100 in M63 medium with 1 mM magnesium sulfate and 0.4% arginine (instead of glucose and CAA). To each well of a 96-well microtiter plate, 100 μl of the diluted culture was added. Microtiter plates were then incubated at 37°C for 24 h in a humidified environment to prevent culture evaporation. To remove unattached bacteria and spent medium, the microtiter dishes were inverted and then washed twice by gently immersing the plate in tap water, followed by removing the liquid by briskly inverting the plate. Microtiter dish biofilms were stained by the addition of 125 μl of 0.1% (wt/vol) crystal violet to each well and incubated for 15 min at room temperature. After the crystal violet solution was removed, the plates were washed three times, as described above, with tap water. Plates were allowed to air dry overnight. The amount of crystal violet retained by each biofilm was measured by adding 150 μl of 30% (vol/vol) glacial acetic acid, incubating the plates for 15 min at room temperature, and mixing the cultures with a pipette. Transfer of 100 μl of this mixture to a 96-well, clear, flat-bottom plate enabled spectrophotometric analysis at 550 nm. Each assay included 4 measurements (technical replicates), which were averaged, and the experiment was performed 5 times (biological replicates). The strains used in these assays are shown in Table S1 in the supplemental material. P. aeruginosa strains PAO1 and PA14 were initially described in references 50 and 51, respectively. All clinical and environmental P. aeruginosa isolates were from the International *Pseudomonas* Consortium Database (IPCD) (43). These strains have been both phenotypically and genotypically characterized (44, 45).

10.1128/mBio.02644-19.7 TABLE S1 Strains used in the crystal violet biofilm assays. The collection of isolates was described in reference 43. CF, cystic fibrosis; ICU, intensive care unit. Download Table S1, DOCX file, 0.02 MB. Copyright © 2020 Lee et al. 2020 Lee et al. This content is distributed under the terms of the Creative Commons Attribution 4.0 International license.

### Flow cell experiments and data acquisition.

Flow cells were prepared and inoculated as previously described (8), with the following modifications. Flow cells were purchased from two sources: the Department of Systems Biology, Technical University of Denmark, and Ibidi (sticky-Slide *VI*^0.4^ with a glass coverslip). An in-line injection port (Ibidi) was used at the inlet for inoculating bacteria into the flow cell. For Ibidi flow cells, elbow connectors (Ibidi) were used to connect the chamber with tubing. The diluted bacterial culture was injected into the flow cell and allowed to incubate for 10 to 20 min without flow on the heating stage at 30°C. Flow was then started at 3 ml/h for the entire acquisition time.

Images were taken using either an Andor iXon electron-multiplying charge-coupled-device (EMCCD) camera with Andor IQ software on an Olympus IX81 microscope equipped with a Zero Drift Correction autofocus system or an Andor Neo scientific complementary metal–oxide–semiconductor (sCMOS) camera with Andor IQ software on an Olympus IX83 microscope equipped with a Zero Drift Correction 2 continuous autofocus system. Bright-field images were taken every 3 s (30-ms exposure time) on the IX81 system and every 100 ms (30-ms exposure time) on the IX83 system. For flagellum staining experiments, bright-field images were taken every 3 s (30-ms exposure time) on the IX81 system, and two fluorescence images (+0 and +1 μm above the imaging focal plane) were taken every 15 min (100-ms exposure time) using a Lambda LS (Sutter Instrument) xenon arc lamp and a green fluorescent protein (GFP) filter. On the IX81 system, the total acquisition time was ∼40 h, resulting in ∼48,000 images. On the IX83 system, the total acquisition time was ∼20 h, resulting in 720,000 images. The image size was 67 μm by 67 μm (1,024 by 1,024 pixels).

### Multigenerational-family tracking analysis.

Image analysis, family tracking and manual validation, family tree plotting, and tree asymmetry calculations were performed in MATLAB as previously described (8), without modification. Fluorescence images were processed as follows to reduce noise and background signals and enhance flagellum signals. Bandpass filtering, gamma correction, intensity percentile normalization, and then a green color map were applied to the images. Fluorescence images were then laid on top of bright-field images using the lighten opacity setting. Probability distributions were obtained from the family trees as follows. The experimental probability distribution, *P_n_*(*t*), is a two-dimensional (2D) matrix, where the columns represent *n* (the number of cells present in one family) and each row is a time step, *t* (the experimental image data acquisition interval, either every 3 s or 100 ms, depending on the data). For each time step, *t* (in terms of lineage time, with each family starting at a *t*_lineage_ of 0), we keep track of how many families have *n* equal to 0 cells, 1 cell, 2 cells, and so on. The proportion of families with *n* equal to 0, 1, 2, … cells then becomes one row in the matrix. This is equivalent to generating a histogram for *X_t_*(ω) using the states Σ as the bins. The full matrix is generated by repeating this for all time steps in the experimental data. Experimental moments were calculated by equation 14 below. MATLAB functions from the base installation of MATLAB R2015a, MATLAB toolboxes (Statistics and Machine Learning Toolbox, Curve Fitting Toolbox, Image Processing Toolbox, and Signal Processing Toolbox), and custom MATLAB functions were used for all analyses. In particular, the MATLAB functions “fit,” “fmincon,” and “ode45” were used for function fitting, nonlinear least-squares minimization with constraints, and numerical integration.

### Divide-detach stochastic model equations.

Explanation of the model is given in the main text. The solution for the master equation (equation 1), which is the model probability distribution *P_n_*(*t*), is given by

(2) $$\begin{array}{l} P_{n}\left(t\right)=\{\begin{array}{c} 1-\frac{\text{exp}\left[-\rho \left(t\right)\right]}{W\left(t\right)},n=0\\ \frac{\text{exp}\left[-\rho \left(t\right)\right]}{W{\left(t\right)}^{2}}{\left[1-\frac{1}{W\left(t\right)}\right]}^{n-1},n>1 \end{array},\\ W\left(t\right)=\text{exp}\left[-\rho \left(t\right)\right]\left\{1+\overset{t}{\underset{0}{\int }}\mu \left(\tau \right)\text{exp}\left[\rho \left(\tau \right)\right]d\tau \right\},\\ \rho \left(t\right)=\overset{t}{\underset{0}{\int }}\left[\mu \left(\tau \right)-\lambda \left(\tau \right)\right]d\tau , \end{array}$$

and λ(*t*) and μ(*t*) are the single-cell division and detachment rates, respectively. Both rates are functions of time and positive [i.e., λ(*t*) > 0 and μ(*t*) > 0].

The first and second model moments are given by the following equations and by the linear forms of the rates; λ(*t*) = *L*_0_ + *L*_1_*t* and μ(*t*) = *C*_0_ + *C*_1_*t*, which are used for fitting the experimental moments

(3) $$〈n\left(t\right)〉=\text{exp}\left[\left(L_{0}-C_{0}\right)t-\frac{1}{2}\left(C_{1}-L_{1}\right)t^{2}\right],$$

(4) $$〈n{\left(t\right)}^{2}〉=\text{exp}\left[2\left(L_{0}-C_{0}\right)t-\left(C_{1}-L_{1}\right)t^{2}\right]\left\{\begin{array}{c} 1+\left(L_{0}+C_{0}\right)\overset{t}{\underset{0}{\int }}\text{exp}\left[-\left(L_{0}-C_{0}\right)\tau +\frac{1}{2}\left(C_{1}-L_{1}\right)\tau^{2}\right]d\tau + \end{array}\begin{array}{c} \left(C_{1}+L_{1}\right)\overset{t}{\underset{0}{\int }}\tau \text{ exp}\left[-\left(L_{0}-C_{0}\right)\tau +\frac{1}{2}\left(C_{1}-L_{1}\right)\tau^{2}\right]d\tau \end{array}\right\}.$$

Evaluating the integral analytically in equation 4 depends on the relative signs of {*L*_0_, *L*_1_, *C*_0_, *C*_1_}.

### Analytical solutions to the equations in the stochastic model.

The solution for the master equation (equation 1), which is the model probability distribution *P_n_*(*t*), can be found by using the so-called generating function

(5) $$G\left(z,t\right)=\overset{+\infty }{\underset{n=0}{\sum }}z^{n}P_{n}\left(t\right).$$

By plugging the generating function into equation 1, we obtain

(6) $$\partial_{t}G\left(z,t\right)=\left(1-z\right)\left(\lambda z+\mu \right)\partial_{z}G\left(z,t\right).$$

We can rewrite the previous equation in Ricatti’s form, which reads

(7) $$\frac{\mathrm{dz}}{\mathrm{dt}}=S+\mathrm{Qz}+Rz^{2},$$

where *S* = –μ, *Q* = λ + μ, and *R* = –λ. A particular solution of the previous equation is given by *Y*. Then the previous equation can be solved by quadrature [*z*(*t*) = *x*(*t*) + *Y*(*t*)] and

(8) $$\frac{\mathrm{dx}}{\mathrm{dt}}=\left(S+2YQ\right)x+Qz^{2}.$$

A change of variables [*u* = 1/*x* = 1/(*z* – *Y*)] yields

(9) $$\frac{\mathrm{du}}{\mathrm{dt}}=\left(S+2YQ\right)u+Q.$$

The solution of Ricatti’s equation (52) is a homographic function

(10) $$u=C\text{ exp}\left[\int^{}\left(S+2YQ\right)dt\right]+U,$$

where *C* is an arbitrary constant and *U* is a particular solution. We can rewrite *u* as equal to *C*ψ + ϕ, and the solution for *z* reads

(11) $$z=Y+\frac{1}{C\psi +\phi }=\frac{C\psi Y+\phi Y}{C\psi +\phi }=\frac{C\alpha +\beta }{C\gamma +\delta }.$$

By using Palm’s formulae (31–33, 35, 53), it is possible to find *P*_0_(*t*) and *P_n_*(*t*) as a function of η*_t_* and ξ*_t_*, two unknown functions

(12) $$P_{0}\left(t\right)=\xi_{t}\text{ and }P_{n}\left(t\right)=\left[1-P_{0}\left(t\right)][1-\eta_{t}\right]\eta_{t}^{n-1}.$$

By means of geometric series, the generating function *G*(*z*,*t*) reads

(13) $$G\left(z,t\right)=\frac{\xi_{t}+\left(1-\xi_{t}-\eta_{t}\right)z}{1-\eta_{t}z}.$$

By plugging this equation back into equation 6, we can find $\xi_{t}$ and $\eta_{t}$ and finally the solution (equation 2).

To compare the model with the experimental results, we use the model moments defined as

(14) $$〈n{\left(t\right)}^{k}〉=\overset{+\infty }{\underset{n=0}{\sum }}n^{k}P_{n}\left(t\right).$$

From the master equation (equation 1), we can find

(15) $$\frac{d}{\mathrm{dt}}〈n{\left(t\right)}^{k}〉=\overset{+\infty }{\underset{n=0}{\sum }}n^{k}\frac{d}{\mathrm{dt}}P_{n}\left(t\right)=\overset{+\infty }{\underset{n=0}{\sum }}\left\{\left[{\left(n+1\right)}^{k}-n^{k}\right]\lambda \left(t\right)-\left[n^{k}-{\left(n-1\right)}^{k}\right]\mu \left(t\right)\right\}nP_{n}\left(t\right).$$

The first moment reads

(16) $$\begin{array}{l} \frac{d}{\mathrm{dt}}〈n\left(t\right)〉=\underset{n}{\sum }n\frac{d}{\mathrm{dt}}P_{n}\left(t\right)\\ =-\left(\lambda +\mu \right)\underset{n}{\sum }n^{2}P_{n}\left(t\right)+\mu \underset{n}{\sum }\left(n^{2}+n\right)P_{n+1}\left(t\right)+\lambda \underset{n}{\sum }\left(n^{2}-n\right)P_{n-1}\left(t\right)\\ =\lambda \underset{n=1}{\sum }\left[{\left(n-1\right)}^{2}P_{n-1}\left(t\right)+\left(n-1\right)P_{n-1}\left(t\right)\right]+\mu \underset{n=0}{\sum }\left[{\left(n+1\right)}^{2}P_{n+1}\left(t\right)-\left(n+1\right)P_{n+1}\left(t\right)\right]\\ \;-\;(\lambda +\mu )\underset{n=0}{\sum }n^{2}P_{n}\left(t\right)\\ \Leftrightarrow \frac{d}{\mathrm{dt}}〈n\left(t\right)〉=\lambda \left[〈n{\left(t\right)}^{2}〉+〈n\left(t\right)〉\right]+\mu \left[〈n{\left(t\right)}^{2}〉-〈n\left(t\right)〉\right]-\left(\lambda +\mu \right)〈n{\left(t\right)}^{2}〉\\ \Rightarrow \frac{d}{\mathrm{dt}}〈n\left(t\right)〉=\left(\lambda -\mu \right)〈n\left(t\right)〉. \end{array}$$

The solution to this differential equation is

(17) $$〈n\left(t\right)〉=n\left(0\right)\text{exp}\left(-\rho \right),n\left(0\right)=1,\rho \left(t\right)=\overset{t}{\underset{0}{\int }}\left[\mu \left(\tau \right)-\lambda \left(\tau \right)\right]d\tau .$$

Plugging in the linear form of the rates [λ(t) = *L*_0_ + *L*_1_*t* and μ(*t*) = *C*_0_ + *C*_1_*t* ] yields equation 3. The second moment [again, using the linear forms of the rates, λ(t) = *L*_0_ + *L*_1_*t* and μ(*t*) = *C*_0_ + *C*_1_*t*] reads

(18) $$\begin{array}{l} \frac{d}{\mathrm{dt}}〈n{\left(t\right)}^{2}〉=\overset{+\infty }{\underset{n=0}{\sum }}\left\{\left[{\left(n+1\right)}^{2}-n^{2}\right]\left(L_{0}+L_{1}t\right)-\left[n^{2}-{\left(n-1\right)}^{2}\right]\left(C_{0}+C_{1}t\right)\right\}nP_{n}\left(\text{t}\right)\\ =\;〈n\left(t\right)〉\left(L_{0}+L_{1}t+C_{0}+C_{1}t\right)+2〈n{\left(t\right)}^{2}〉(L_{0}+L_{1}t-C_{0}-C_{1}t). \end{array}$$

Elementary computations yield equation 4.

### Hydrodynamic model of TFP retraction during flagellum-mediated spinning.

We adapt the hydrodynamic model that we developed previously (37) to investigate the effects of TFP on flagellum-mediated spinning. Here, we consider a bacterium consisting of a cylindrical body attached to the surface at the pole opposite the flagellum, a helical filament for the flagellum (with a length equal to that of the body), and a straight filament two-thirds of the body’s length for a pilus protruding from the body. We use resistive-force theory (54) to relate the angular velocities of each component of the bacterium to the torques from the flagellar motor, the viscous resistance from the fluid, and the flagellar hook, which resists bending between the head and the flagellum. The resultant model is used to consider how TFP affect the angle that the bacterium makes with the surface during flagellum-mediated spinning and thereby how they “stand up” to a nearly vertical orientation commonly observed before detachment.

We use the example where the ratio of hook stiffness to motor torque is 0.5 to show the effects of TFP (see reference 37 for details of the stiffness/motor torque ratio). We show time using units of seconds and a torque value of 2 pN μm. We observe two significant effects on the surface angle when TFP retract during spinning: (i) the bacterium decreases its surface angle after retraction and (ii) the amplitude of oscillations in surface angle decreases after retraction. The opposite occurs when TFP extend during spinning: (i) the bacterium increases its surface angle after extension and (ii) the amplitude of oscillations in surface angle increases after extension. The strength of these effects depends on the choice of value of the flagellar motor torque and hook stiffness in the hydrodynamic model.

### TFP and flagellum activity metrics.

To characterize appendage activity during family tree tracking, we use the algorithms previously described (8) for TFP activity and adapt them for flagellum activity. As previously described, TFP activity is inferred by recognizing surface translational motion, which is the predominant behavior for TFP-driven motion for P. aeruginosa cells that attach to the surface during early biofilm development. Analogous to this is that the most common mode of flagellum activity is surface-attached “spinning,” where cells attach via one pole on the surface and spin at angular velocities consistent with typical flagellum motor output (∼5 rad/s) (36, 37). So, flagellum activity is inferred by recognizing surface rotational motion. Based on the majority of flagellum-mediated surface spinning behavior producing trajectories that are tightly clustered together and have strongly subdiffusive mean squared displacements (MSDs), the multiparameter metric for flagellum activity is defined as follows. A bacterium has flagellum activity during a given time point when it is spinning and has non-zero displacement over a *w* frame-moving window every *w*/10 frames. A cell that is spinning is defined as having the following characteristics during the *w* frame window: it has an MSD slope of less than 0.9, and the maximum 2-point distance of its trajectory is greater than or equal to 15% of its maximum cell body length and less than its cell body length. As previously described, the value for *w* was 100 (8).

10.1128/mBio.02644-19.8 TEXT S1 Supplemental references. Download Text S1, DOCX file, 0.02 MB. Copyright © 2020 Lee et al. 2020 Lee et al. This content is distributed under the terms of the Creative Commons Attribution 4.0 International license.
