# Supplementary material for: Social Cooperativity of Bacteria during Reversible Surface Attachment in Young Biofilms: a Quantitative Comparison of Pseudomonas aeruginosa PA14 and PAO1
Source: mBio. 2020 Feb 25;11(1):e02644-19. doi: 10.1128/mBio.02644-19 (PMC7042694; doi:10.1128/mBio.02644-19)
Supplement: TABLE S1 [file mBio.02644-19-st001.docx]

| **Strain ID** | **Source ID** | **Local Strain collection #** | **Origin and source** | **Original reference** |
| --- | --- | --- | --- | --- |
| PA14 WT |  | DH123 | PA14 *P. aeruginosa* wild type | (51) |
| PAO1 WT |  | DH1467 | PAO1 *P. aeruginosa* wild type | (55) |
| 1268 | 15108-1 | DH3446 | ICU (acute infection), Spain | (56) |
| 87 | 679 | DH3418 | Non CF Urine sample, male, Wroclaw Poland, 2011 | (43) |
| 95 | CPHL9433 | DH3425 | Tobacco plant, Philippines | (57) |
| 1103 | AUS23 | DH3436 | Adult CF (2007), Brisbane, Australia | (58) |
| 80 | AMT0060-1 | DH3411 | Pediatric CF, Seattle, WA | (59) |
| 1273 | TBCF10839 | DH3451 | CF, Germany | (60) |
| 1260 | AMT0023-30 | DH3441 | Pediatric CF, Seattle, WA | (59) |
| 94 | U018A | DH3424 | Hobart, Australia, CF patient | (57) |
| 92 | LMG14084 | DH3422 | Bucharest, Romania, Water, 1960-1964 | (57) |
| 85 | IST27N | DH3416 | Lisbon Portugal, CF patient | (61) |
| 1259 | AA2 | DH3440 |  |  |
| 93 | Pr335 | DH3423 | Prague, Czech Republic, Hospital environment 1997 | (57) |
| 91 | Jpn1563 | DH3421 | Lake Tamaco, Japan, Lake water, 2003 | (57) |
| 84 | IST27 | DH3415 | Lisbon Portugal, CF patient | (61) |
| 1258 | AUS52 | DH3439 | Adult CF (2008), Hobart, Australia | (62, 63) |
| 1266 | Mi162-2 | DH3445 | Non CF burn, Ann Arbor, MI, 1997 | (57) |
| 89 | 1709-12 | DH3420 | Leuven Belgium Non CF clinical 2004 | (57) |
| 2495 | LES400 | DH3459 | CF, U.K. | (64) |
| 1272 | A5803 | DH3450 | Community-acquired pneumonia | (65) |
| 1264 | 39016 | DH3444 | Keratitis eye isolate, U.K. | (66) |
| 88 | NH57388A | DH3419 | CF, Denmark | (67) |
| 2496 | LES431 | DH3460 | Non CF parent of CF patient, U.K. | (66) |
| 1271 | KKI | DH3449 | CF, Germany | (68) |
| 1262 | CHA | DH3443 | CF | (69) |
| 83 | PAK | DH3414 | Clinical non CF | (70) |
| 2617 | LESB58 | DH3461 | CF, U.K., 1988 | (71) |
| 1270 | 39177 | DH3448 | Keratitis, Manchester U.K. | (66) |
| 1261 | AMT0023-34 | DH3442 | Pediatric CF, Seattle, WA | (59) |
| 82 | AMT0060-3 | DH3413 | Pediatric CF, Seattle, WA | (59) |
| 2045 | UCBPP-PA14 | DH3458 | Human Burn isolate | (51) |
| 1269 | 13121-1 | DH3447 | ICU (acute infection), France | (65) |
| 1256 | C3719 | DH3437 | CF, Manchester, U.K. | (72) |
| 81 | AMT0060-2 | DH3412 | Pediatric CF, Seattle, WA | (59) |
