# Supplementary material for: Social Cooperativity of Bacteria during Reversible Surface Attachment in Young Biofilms: a Quantitative Comparison of Pseudomonas aeruginosa PA14 and PAO1
Source: mBio. 2020 Feb 25;11(1):e02644-19. doi: 10.1128/mBio.02644-19 (PMC7042694; doi:10.1128/mBio.02644-19)
Supplement: TEXT S1 [file mBio.02644-19-s0001.docx]

# Supplementary Materials

## Supplementary References

8. Lee CK, de Anda J, Baker AE, Bennett RR, Luo Y, Lee EY, Keefe JA, Helali JS, Ma J, Zhao K, Golestanian R, O’Toole GA, Wong GCL. 2018. Multigenerational memory and adaptive adhesion in early bacterial biofilm communities. Proceedings of the National Academy of Sciences 115:4471-4476.

42. Freschi L, Jeukens J, Kukavica-Ibrulj I, Boyle B, Dupont M-J, Laroche J, Larose S, Maaroufi H, Fothergill JL, Moore M, Winsor GL, Aaron SD, Barbeau J, Bell SC, Burns JL, Camara M, Cantin A, Charette SJ, Dewar K, Déziel É, Grimwood K, Hancock REW, Harrison JJ, Heeb S, Jelsbak L, Jia B, Kenna DT, Kidd TJ, Klockgether J, Lam JS, Lamont IL, Lewenza S, Loman N, Malouin F, Manos J, McArthur AG, McKeown J, Milot J, Naghra H, Nguyen D, Pereira SK, Perron GG, Pirnay J-P, Rainey PB, Rousseau S, Santos PM, Stephenson A, Taylor V, Turton JF, Waglechner N, et al. 2015. Clinical utilization of genomics data produced by the international *Pseudomonas aeruginosa* consortium. Frontiers in Microbiology 6:1036.

43. De Soyza A, Hall AJ, Mahenthiralingam E, Drevinek P, Kaca W, Drulis-Kawa Z, Stoitsova SR, Toth V, Coenye T, Zlosnik JEA, Burns JL, Sá-Correia I, De Vos D, Pirnay J-P, J. Kidd T, Reid D, Manos J, Klockgether J, Wiehlmann L, Tümmler B, McClean S, Winstanley C, pathogens” EFfCABCsvdocf. 2013. Developing an international *Pseudomonas aeruginosa* reference panel. MicrobiologyOpen 2:1010-1023.

44. Cullen L, Weiser R, Olszak T, Maldonado RF, Moreira AS, Slachmuylders L, Brackman G, Paunova-Krasteva TS, Zarnowiec P, Czerwonka G, Reilly J, Drevinek P, Kaca W, Melter O, De Soyza A, Perry A, Winstanley C, Stoitsova SR, Lavigne R, Mahenthiralingam E, Sá-Correia I, Coenye T, Drulis-Kawa Z, Augustyniak D, Valvano MA, McClean S. 2015. Phenotypic characterization of an international *Pseudomonas aeruginosa* reference panel: strains of cystic fibrosis (CF) origin show less in vivo virulence than non-CF strains. Microbiology 161:1961-1977.

45. Freschi L, Bertelli C, Jeukens J, Moore MP, Kukavica-Ibrulj I, Emond-Rheault J-G, Hamel J, Fothergill JL, Tucker NP, McClean S, Klockgether J, de Soyza A, Brinkman FSL, Levesque RC, Winstanley C. 2018. Genomic characterisation of an international *Pseudomonas aeruginosa* reference panel indicates that the two major groups draw upon distinct mobile gene pools. FEMS Microbiology Letters 365.

51. Rahme LG, Stevens EJ, Wolfort SF, Shao J, Tompkins RG, Ausubel FM. 1995. Common virulence factors for bacterial pathogenicity in plants and animals. Science 268:1899-1902.

55. Stover CK, Pham XQ, Erwin AL, Mizoguchi SD, Warrener P, Hickey MJ, Brinkman FS, Hufnagle WO, Kowalik DJ, Lagrou M, Garber RL, Goltry L, Tolentino E, Westbrock-Wadman S, Yuan Y, Brody LL, Coulter SN, Folger KR, Kas A, Larbig K, Lim R, Smith K, Spencer D, Wong GK, Wu Z, Paulsen IT, Reizer J, Saier MH, Hancock RE, Lory S, Olson MV. 2000. Complete genome sequence of *Pseudomonas aeruginosa* PAO1, an opportunistic pathogen. Nature 406:959-964.

56. Köhler T, Buckling A, van Delden C. 2009. Cooperation and virulence of clinical *Pseudomonas aeruginosa* populations. Proceedings of the National Academy of Sciences 106:6339-6344.

57. Pirnay J-P, Bilocq F, Pot B, Cornelis P, Zizi M, Van Eldere J, Deschaght P, Vaneechoutte M, Jennes S, Pitt T, De Vos D. 2009. *Pseudomonas aeruginosa* Population Structure Revisited. PLOS ONE 4:e7740.

58. O'Carroll MR, Syrmis MW, Wainwright CE, Greer RM, Mitchell P, Coulter C, Sloots TP, Nissen MD, Bell SC. 2004. Clonal strains of *Pseudomonas aeruginosa* in paediatric and adult cystic fibrosis units. European Respiratory Journal 24:101-106.

59. Mulcahy LR, Burns JL, Lory S, Lewis K. 2010. Emergence of *Pseudomonas aeruginosa* Strains Producing High Levels of Persister Cells in Patients with Cystic Fibrosis. Journal of Bacteriology 192:6191-6199.

60. Bezuidt OK, Klockgether J, Elsen S, Attree I, Davenport CF, Tümmler B. 2013. Intraclonal genome diversity of *Pseudomonas aeruginosa* clones CHA and TB. BMC Genomics 14:416.

61. Leitão JH, Alvim T, Sá-Correia I. 1996. Ribotyping of *Pseudomonas aeruginosa* isolates from patients and water springs and genome fingerprinting of variants concerning mucoidy. Pathogens and Disease 13:287-292.

62. Bradbury R, Champion A, Reid DW. 2008. Poor clinical outcomes associated with a multi-drug resistant clonal strain of *Pseudomonas aeruginosa* in the Tasmanian cystic fibrosis population. Respirology 13:886-892.

63. Kidd TJ, Ramsay KA, Hu H, Marks GB, Wainwright CE, Bye PT, Elkins MR, Robinson PJ, Rose BR, Wilson JW, Grimwood K, Bell SC. 2013. Shared *Pseudomonas aeruginosa* genotypes are common in Australian cystic fibrosis centres. European Respiratory Journal 41:1091-1100.

64. Salunkhe P, Smart CHM, Morgan JAW, Panagea S, Walshaw MJ, Hart CA, Geffers R, Tümmler B, Winstanley C. 2005. A Cystic Fibrosis Epidemic Strain of *Pseudomonas aeruginosa* Displays Enhanced Virulence and Antimicrobial Resistance. Journal of Bacteriology 187:4908-4920.

65. Wiehlmann L, Wagner G, Cramer N, Siebert B, Gudowius P, Morales G, Köhler T, van Delden C, Weinel C, Slickers P, Tümmler B. 2007. Population structure of *Pseudomonas aeruginosa*. Proceedings of the National Academy of Sciences 104:8101-8106.

66. Stewart RMK, Wiehlmann L, Ashelford KE, Preston SJ, Frimmersdorf E, Campbell BJ, Neal TJ, Hall N, Tuft S, Kaye SB, Winstanley C. 2011. Genetic Characterization Indicates that a Specific Subpopulation of *Pseudomonas aeruginosa* Is Associated with Keratitis Infections. Journal of Clinical Microbiology 49:993-1003.

67. Hoffmann N, Rasmussen TB, Jensen P, Stub C, Hentzer M, Molin S, Ciofu O, Givskov M, Johansen HK, Høiby N. 2005. Novel Mouse Model of Chronic *Pseudomonas aeruginosa* Lung Infection Mimicking Cystic Fibrosis. Infection and Immunity 73:2504-2514.

68. Cramer N, Wiehlmann L, Ciofu O, Tamm S, Høiby N, Tümmler B. 2012. Molecular Epidemiology of Chronic *Pseudomonas aeruginosa* Airway Infections in Cystic Fibrosis. PLOS ONE 7:e50731.

69. Toussaint B, Delicattree I, Vignais PM. 1993. *Pseudomonas aeruginosa* Contains an IHF-like Protein That Binds to the *algD* Promoter. Biochemical and Biophysical Research Communications 196:416-421.

70. Totten PA, Lory S. 1990. Characterization of the type a flagellin gene from *Pseudomonas aeruginosa* PAK. Journal of Bacteriology 172:7188-7199.

71. Kukavica-Ibrulj I, Bragonzi A, Paroni M, Winstanley C, Sanschagrin F, O'Toole GA, Levesque RC. 2008. In Vivo Growth of *Pseudomonas aeruginosa* Strains PAO1 and PA14 and the Hypervirulent Strain LESB58 in a Rat Model of Chronic Lung Infection. Journal of Bacteriology 190:2804-2813.

72. Jones AM, Dodd ME, Doherty CJ, Govan JR, Webb AK. 2002. Increased treatment requirements of patients with cystic fibrosis who harbour a highly transmissible strain of *Pseudomonas aeruginosa*. Thorax 57:924-925.
